# Supplementary material for: One step forward, two steps back: Transcriptional advancements and fermentation phenomena in Actinobacillus succinogenes 130Z
Source: PLoS One. 2021 May 3;16(5):e0245407. doi: 10.1371/journal.pone.0245407 (PMC8092802; doi:10.1371/journal.pone.0245407)
Supplement: S1 File — (PDF) [file pone.0245407.s001.pdf]

Supporting Information

Supplementary Tables

Table S1. Genetics parts and sequences.

Genetic Part	Sequence	Source
E. coli origin of replication pBR322 (EC ori)	agctttctgctaatcctgttaccagtggctgctgccagtggcgataagtcgtgtcttaccgggttggactcaagacgatagttaccggataag<br>gcgcagcggtcgggctgaacggggggttcgtgcacacagcccagcttggagcgaacgacctacaccgaactgagatacctacagcgt<br>gagctatgagaaagcgccacgcttcccgaagggagaaaggcggacaggtatccggtaagcggcagggtcggaacaggagagcgca<br>cgagggagcttccagggggaaacgcctggtatctttatagtcctgtcgggtttcgccacctctgacttgagcgtcgatttttgtgatgctcgtc<br>aggggggcggagcctatggaaaaacgccagcaacgcggcctttttacggttcctggccttttgctggccttttgctcacatgttctttcctgc<br>gttatcccctgattctgtggataaccgtattaccgcctttgagtgagctgataccgctcgccgcagccgaacgaccgagcgcagcgagtca<br>gtgagcgaggaacgcgaagagcgcctgatgcgga	Plasmid: PLGZ920 (1)
A. succinogenes origin of replication (AS ori)	aattcactggccgtcgttttacaacgtcgtgactgggaaaaccctggcgttacccaacttaatcgccttgcagcacatccccctttcgccagc<br>tggcgtaatagcgaagaggcccgcaccgatcgcccttcccaacagttgcgcagcctgaatggcgaatggcgccagatgaagcagagc<br>gcgccttcgctgtcctattcaatgtattggggatcgaagcccccgaccgcgaacgccttgccttctatctgcgattggaccctctgacttggg<br>gttgatgttcatgccgcctgtttttcctgctcattggcacgtttcgcaacctgttctcattgcggacaccttttccagcctcgtttggaaagtttcat<br>tgccagtaataccaatgctttagaaagaaaaagggatcgaacttttgacattcgatccctttttctgtaatctgtttcgtgcgttctttgctaagat<br>acagaccctagacaagtcatatcttagcaaagggtagctagtaatgcaagagattgcgaagcgtccctactaccaaaaaaccattcaacga<br>cgtaaacagacaaacgcaaaccttaaattagacggtcttcagctcggacttcggaagaataaacaggcgtagaagtgataacgttcttaata<br>cgaaaattaagctctgtctccgtttcgtgctacggttagaaaggcgaaagccccaagaaatacaagcacacctgataagcgagatttaagg<br>ataacagcgaaattcaatagggtctgaatttccaaactaggttaaatgccacgacgttttattgttgccccattcaagcaacatttgagaaccg<br>aatagaaatcttttagtaaaaagcgttctttttgggtcagcggttaatgtggacggtttaacggtttttcccctgcgggtcgtattggaaagcca<br>ttgaaaagctgatggataactctgcgagttacccacgagctttccaacagctttccaacactaaaaacctaccgcccacaataaccacttcc<br>ctaataataaaattttttttatttttattttggttcaaaggctcacgatgttcgcctaataaaacgaagtcgcctatcggctccgctgatttttatatatc<br>actctcggggcttttggtgtactattgtcttttgtaatagcaaggacacaaaaagggtactcttcgagtttcctttttgaccttgcaaaagggcttt<br>gccccccttgacccccgaccgctttcagcggtcaaaatagaagaacggacaccattatgaaacgtgagaaagagataaaaatcaggctca<br>ccgaaaacgagtatcaagccttgttagagagaaaaacgaaagcaaggcttgcggagtgggttcgggaagttgccctggaacagcaacct<br>aagcgacagccgaaagtaatcgaccctgcgttactgttcgagctgaaccgcataggcgtaaacctgaaccaaatcgcccgacaatgcaa<br>cagtcaaaagccgagcattgaccttgttagcgtgttggcgaccttgcgagaaattgaaaaaaatctcaaaaaattgcgagaattgagcctat<br>gatcgttaaatttttaagaaacacggtaagggaaaagctagtagttgcaaggcttgcgtggactatttactaaataagcctgacgacaccgc<br>ccaaatcctgca	Plasmid: PLGZ920 (1)

Kanamycin resistance (*kanR*)	atgattgaacaagatggattgcacgcaggttctccggccgcttgggtggagaggctattcggctatgactgggcacaacagacaatcggc<br>tgctctgatgccgccgtgttccggctgtcagcgcaggggcgcccggttctttttgtcaagaccgacctgtccggtgccctgaatgaactgc<br>aggacgaggcagcgcggctatcgtggctggccacgacgggcgttccttgcgcagctgtgctcgacgttgtcactgaagcgggaaggga<br>ctggctgctattgggcgaagtgccggggcaggatctcctgtcatctcaccttgctcctgccgagaaagtatccatcatggctgatgcaatgc<br>ggcggctgcatacgcttgatccggctacctgcccattcgaccaccaagcgaaacatcgcatcgagcgagcacgtactcggatggaagcc<br>ggtcttgtcgatcaggatgatctggacgaagagcatcaggggctcgcgccagccgaactgttcgccaggctcaaggcgcgcatgcccg<br>acggcgaggatctcgtcgtgacccatggcgatgcctgcttgccgaatatcatggtggaaaatggccgcttttctggattcatcgactgtggc<br>cggctgggtgtggcggaccgctatcaggacatagcgttggctacccgtgatattgctgaagagcttggcggcgaatgggctgaccgcttc<br>ctcgtgctttacggtatcgccgctcccgattcgcagcgcatcgccttctatcgccttcttgacgagttcttctga	Plasmid: pBBR1MCS-2 (2)
Green fluorescent protein (*gFPuv*)	atgagtaaaggagaagaacttttcactggagttgtcccaattcttgttgaattagatggtgatgttaatgggcacaaattttctgtcagtggaga<br>gggtgaaggtgatgcaacatacggaaaacttacccttaaatttatttgcactactggaaaactacctgttccgtggccaacacttgtcactact<br>ttctcttatggtgttcaatgcttttcccgttatccggatcacatgaaacggcatgactttttcaagagtgccatgcccgaaggttatgtacagga<br>acgcactatatctttcaaagatgacgggaactacaagacgcgtgctgaagtcaagtttgaaggtgatacccttgttaatcgtatcgagttaaa<br>aggtattgattttaaagaagatggaaacattctcggacacaaactggagtacaactataactcacacaatgtatacatcacggcagacaaac<br>aaaagaatggaatcaaagctaacttcaaaattcgccacaacattgaagatggctccgttcaactagcagaccattatcaacaaaatactcca<br>attggcgatggccctgtccttttaccagacaaccattacctgtccacacaatctgccctttcgaaagatcccaacgaaaagcgtgaccacat<br>ggtccttcttgagtttgtaactgctgctgggattacacatggcatggatgagctctacaaataa	Plasmid: pBbB7a-GFP (3)
Flavin binding fluorescent protein (*fbfp*)	atgagaggatcgcatcaccatcaccatcacggatccatgatcaacgcaaaactcctgcaactgatggtcgaacattccaacgatggcatcg<br>ttgtcgccgagcaggaaggcaatgagagcatccttatctacgtcaacccggccactgagcgcctgaccggctactgcgccgacgatattc<br>tctatcaggacgcacgttttcttcagggcgaggatcacgaccagccgggcatcgcaattatccgcgaggcgatccgcgaaggccgcccc<br>tgctgccaggtgctgcgcaactaccgcaaagacggcagcctgttctggaacgagttgtccatcacaccggtgcacaacgaggcggacc<br>agctgacctactacatcggcatccagcgcgatgtcacagcgcaagtattcgccgaggaaagggttcgcgagctggaggctgaagtggc<br>ggaactgcgccggcagcagggccaggccaagcactga	Synthesized from known sequence (4))<br>(Thermo Fisher Scientific)
lacI	gtgaaaccagtaacgttatacgatgtcgcagagtatgccggtgtctcttatcagaccgtttcccgcgtggtgaaccaggccagccacgtttc<br>tgcgaaaacgcgggaaaaagtggaagcggcgatggcggagctgaattacattcccaaccgcgtggcacaacaactggcgggcaaaca<br>gtcgttgctgattggcgttgccacctccagtctggccctgcacgcgccgtcgcaaattgtcgcggcgattaaatctcgcgccgatcaactg<br>ggtgccagcgtggtggtgtcgatggtagaacgaagcggcgtcgaagcctgtaaagcggcggtgcacaatcttctcgcgcaacgcgtca<br>gtgggctgatcattaactatccgctggatgaccaggatgccattgctgtggaagctgcctgcactaatgttccggcgttatttcttgatgtctct<br>gaccagacacccatcaacagtattattttctcccatgaagacggtacgcgactgggcgtggagcatctggtcgcattgggtcaccagcaaa<br>tcgcgctgttagcgggcccattaagttctgtctcggcgcgtctgcgtctggctggctggcataaatatctcactcgcaatcaaattcagccga<br>tagcggaacgggaaggcgactggagtgccatgtccggttttcaacaaaccatgcaaatgctgaatgagggcatcgttcccactgcgatgc<br>tggttgccaacgatcagatggcgctgggcgcaatgcgcgccattaccgagtccgggctgcgcgttggtgcggacatctcggtagtggga<br>tacgacgataccgaagacagctcatgttatatcccgccgttaaccaccatcaaacaggattttcgcctgctggggcaaaccagcgtggacc<br>gcttgctgcaactctctcagggccaggcggtgaagggcaatcagctgttgcccgtctcactggtgaaaagaaaaaccaccctggcgccc<br>aatacgcaaaccgcctctccccgcgcgttggccgattcattaatgcagctggcacgacaggtttcccgactggaaagcgggcagtga	*E. coli* MG1655 purchased from Yale's Coli Genetic Stock Center https://cgsc.biology.yale.edu/

Table S2. Promoter library.

Promoter Name		Sequence	Source
P_{lac} (core sequence bolded) with flanking operator regions		ggcagtgagcgcaacgcaattaatgtgagttagctcactcattaggcaccccaggc**ttt** **acactttatgcttccggctcgtatgttgtgtgg**aattgtgagcggataacaa	*E. coli* MG1655 gDNA
P_{pcka}		tcgataaattgaaaatgcagcaatagaggaaacacggtttgtttgagtgaaaacagccg tgtttttcatttaccgccataaaaatttgaaacggatcacaaatcatgaaaaaaatacgttc aaattagaactaattatcgaaaatttgatctagttaacatttttaggtataaatagttttaaaa tagatctagtttggatttttaattttaaattatcaatgaggtga	*A. succinogenes* gDNA
Anderson promoters	BBa_J23100	ttgacggctagctcagtcctaggtacagtgctagc	iGEM Registry of Standard Biological Parts(Anderson, 2008)
	BBa_J23101	tttacagctagctcagtcctaggtattatgctagc	
	BBa_J23102	ttgacagctagctcagtcctaggtactgtgctagc	
	BBa_J23103	ctgatagctagctcagtcctagggattatgctagc	
	BBa_J23104	ttgacagctagctcagtcctaggtattgtgctagc	
	BBa_J23105	tttacggctagctcagtcctaggtactatgctagc	
	BBa_J23106	tttacggctagctcagtcctaggtatagtgctagc	
	BBa_J23107	tttacggctagctcagccctaggtattatgctagc	
	BBa_J23108	ctgacagctagctcagtcctaggtataatgctagc	
	BBa_J23109	tttacagctagctcagtcctagggactgtgctagc	
	BBa_J23110	tttacggctagctcagtcctaggtacaatgctagc	
	BBa_J23111	ttgacggctagctcagtcctaggtatagtgctagc	
	BBa_J23112	ctgatagctagctcagtcctagggattatgctagc	
	BBa_J23113	ctgatggctagctcagtcctagggattatgctagc	
	BBa_J23114	tttatggctagctcagtcctaggtacaatgctagc	
	BBa_J23115	tttatagctagctcagcccttggtacaatgctagc	
	BBa_J23116	ttgacagctagctcagtcctagggactatgctagc	
	BBa_J23117	ttgacagctagctcagtcctagggattgtgctagc	
	BBa_J23118	ttgacggctagctcagtcctaggtattgtgctagc	
	BBa_J23119	ttgacagctagctcagtcctaggtataatgctagc	

Table S3. Primers for plasmid construction.

Primer Pair	Target(s)	Promoter Variant on Primer Tails	Template	Sequences	
AS001	EC ori, AS ori, P_{lac}, *gFPuv*		AS-plac(c)-GFPuv-amp	Forward	cgaaaagtgccacctgacg
				Reverse	gtaactgtcagaccaagtttacgagc
AS002	*kanR*		pBR1MCS-2(Kovach et al., 1995)	Forward	agctcgtaaacttggtctgacagttaccgcttggtcggtcatttcg
				Reverse	ggacgtcaggtggcacttttcggcagtgggcttacatggcgatag
AS003	EC ori, AS ori, *gFPuv*, *kanR*	BBa_J23100	SSBIO-AS001	Forward	ctaggtacagtgctagcaaattcaaaagatcttttaagaaggagatatacatatgag
				Reverse	gactgagctagccgtcaaggaaacctgtcgtggacgtc
AS004		BBa_J23101		Forward	ctaggtattatgctagcaaattcaaaagatcttttaagaaggagatatacatatgag
				Reverse	gactgagctagctgtaaaggaaacctgtcgtggacgtc
AS005		BBa_J23102		Forward	ctaggtactgtgctagcaaattcaaaagatcttttaagaaggagatatacatatgag
				Reverse	gactgagctagctgtcaaggaaacctgtcgtggacgtc
AS006		BBa_J23103		Forward	ctagggattatgctagcaaattcaaaagatcttttaagaaggagatatacatatgag
				Reverse	gactgagctagctatcagggaaacctgtcgtggacgtc
AS007		BBa_J23104		Forward	ctaggtattgtgctagcaaattcaaaagatcttttaagaaggagatatacatatgag
				Reverse	gactgagctagctgtcaaggaaacctgtcgtggacgtc
AS008		BBa_J23105		Forward	ctaggtactatgctagcaaattcaaaagatcttttaagaaggagatatacatatgag
				Reverse	gactgagctagccgtaaaggaaacctgtcgtggacgtc
AS009		BBa_J23106		Forward	ctaggtatagtgctagcaaattcaaaagatcttttaagaaggagatatacatatgag
				Reverse	gactgagctagccgtaaaggaaacctgtcgtggacgtc
AS010		BBa_J23107		Forward	ctaggtattatgctagcaaattcaaaagatcttttaagaaggagatatacatatgag
				Reverse	ggctgagctagccgtaaaggaaacctgtcgtggacgtc
AS011		BBa_J23108		Forward	ctaggtataatgctagcaaattcaaaagatcttttaagaaggagatatacatatgag
				Reverse	gactgagctagctgtcagggaaacctgtcgtggacgtc
AS012		BBa_J23109		Forward	ctagggactgtgctagcaaattcaaaagatcttttaagaaggagatatacatatgag
				Reverse	gactgagctagctgtaaaggaaacctgtcgtggacgtc
AS011		BBa_J23110		Forward	ctaggtacaatgctagcaaattcaaaagatcttttaagaaggagatatacatatgag
				Reverse	gactgagctagccgtaaaggaaacctgtcgtggacgtc
AS012		BBa_J23111		Forward	ctaggtatagtgctagcaaattcaaaagatcttttaagaaggagatatacatatgag
				Reverse	gactgagctagccgtcaaggaaacctgtcgtggacgtc

AS013		BBa_J23112		Forward	ctagggattatgctagcaaattcaaaagatcttttaagaaggagatatacatatgag
				Reverse	gactgagctagctatcagggaaacctgtcgtggacgtc
AS014		BBa_J23113		Forward	ctagggattatgctagcaaattcaaaagatcttttaagaaggagatatacatatgag
				Reverse	gactgagctagccatcagggaaacctgtcgtggacgtc
AS015		BBa_J23114		Forward	ctaggtacaatgctagcaaattcaaaagatcttttaagaaggagatatacatatgag
				Reverse	gactgagctagccataaaggaaacctgtcgtggacgtc
AS016		BBa_J23115		Forward	cttggtacaatgctagcaaattcaaaagatcttttaagaaggagatatacatatgag
				Reverse	ggctgagctagctataaaggaaacctgtcgtggacgtc
AS017		BBa_J23116		Forward	ctagggactatgctagcaaattcaaaagatcttttaagaaggagatatacatatgag
				Reverse	gactgagctagctgtcaaggaaacctgtcgtggacgtc
AS018		BBa_J23117		Forward	ctagggattgtgctagcaaattcaaaagatcttttaagaaggagatatacatatgag
				Reverse	gactgagctagctgtcaaggaaacctgtcgtggacgtc
AS019		BBa_J23118		Forward	ctaggtattgtgctagcaaattcaaaagatcttttaagaaggagatatacatatgag
				Reverse	gactgagctagccgtcaaggaaacctgtcgtggacgtc
AS020		BBa_J23119		Forward	ctaggtataatgctagcaaattcaaaagatcttttaagaaggagatatacatatgag
				Reverse	gactgagctagctgtcaaggaaacctgtcgtggacgtc
AS021	P_{pcka}		PLGZ920(Kim et al., 2004)	Forward	tcgataaattgaaaatgcagcaatagaggaaacac
				Reverse	tcacctcattgataatttaaaattaaaaatccaaactaga
AS022	EC ori, AS ori, *gFPuv*, *kanR*		SSBIO-AS001	Forward	ttaaattatcaatgaggtgaaattcaaaagatcttttaagaaggagatatacatatgag
				Reverse	gcattttcaatttatcgaggaaacctgtcgtggacgtc
AS023	EC ori, AS ori, *kanR*, primer variant		SSBIO-AS001	Forward	cagccgatagtctggaacagccgtcaacataacctcggacag
				Reverse	gtgatggtgatgcgatcctctcatatgtatatctccttcttaaaagatcttttg
AS024	EC ori, AS ori, P_{lac}, *gFPuv*, *lacI*		AS-plac(i)-GFPuv-amp	Forward	same as AS001 forward
				Reverse	agttccagactatcggctgtagcc
AS025				Forward	agccgatagtctggaacttggtaactgtcagaccaagtttactc
				Reverse	same as AS002 reverse
AS026	EC ori, AS ori, *gFPuv*, *lacI*, P_{lac} flanking regions		SSBIO-AS002	Forward	tcctaggtacagtgctagcaattgtgagcggataacaatttcatttcagaattc
				Reverse	ggctgatgcaatgcggcgg
AS027				Forward	atgatggatactttctcggcaggagcaaggtg
				Reverse	ctgagctagccgtcaangcctggggtgcctaatgagtgagctaactc

Table S4. Plasmids used in this study.

Template Plasmid	Relevant Components	Source
AS-Plac(c)-GFPuv-amp	EC ori, AS ori, P_{lac}, *gFPuv*,	Unpublished, Saha Lab at UNL (Lincoln, NE)
pBR1MCS-2	*kanR*	Addgene 85168 (Watertown, MA)
PLGZ920	P_{pcka}	Generous donation form Dr. Gregg Beckham at the National Renewable Energy Lab (Golden, CO)
AS-Plac(i)-GFPuv-amp	EC ori, AS ori, *lacI*, P_{lac}, *gFPuv*	Unpublished, Saha Lab at UNL (Lincoln, NE)
**Constructed Plasmid**	**Components**	**Source**
SSBIO-AS001	EC ori - AS ori - constitutive promoter library - *gFPuv* - *kanR*	This study
SSBIO-AS002	EC ori - AS ori - constitutive promoter variant - *fbfp* - *kanR*	
SSBIO-AS003	EC ori - AS ori – *lacI* - P_{lac} - *gFPuv* - *kanR*	
SSBIO-AS004	EC ori - AS ori – *lacI* – *BBa_J23100* - *gFPuv* - *kanR*	

Table S5. Strains used in this study.

Strain	Vector	Promoter variant	Source
Actinobacillus succinogenes 130Z (ATCC 55618)			American Type Culture Collection (Manassas, Virginia)
Escherichia coli strain DH10B (NEB #C3019)			New England Biolabs (Ipswich, Massachusetts)
sAS100	SSBIO-AS001	BBa_J23100	This study
sAS101		BBa_J23101	
sAS102		BBa_J23102	
sAS103		BBa_J23103	
sAS104		BBa_J23104	
sAS105		BBa_J23105	
sAS106		BBa_J23106	
sAS107		BBa_J23107	
sAS108		BBa_J23108	
sAS109		BBa_J23109	
sAS110		BBa_J23110	
sAS111		BBa_J23111	
sAS112		BBa_J23112	
sAS113		BBa_J23113	
sAS114		BBa_J23114	
sAS115		BBa_J23115	
sAS116		BBa_J23116	
sAS117		BBa_J23117	
sAS118		BBa_J23118	
sAS119		BBa_J23119	
sAS120		P_{lac}	
sAS121		P_{pcka}	
sAS122	SSBIO-AS002	P_{pcka}	
sAS123		BBa_J23114	
sAS124	SSBIO-AS003	P_{lac}	
sAS125	SSBIO-AS004	C-Bba_J23100	
sAS126		G-Bba_J23100	
sAS127		T-Bba_J23100	
sAS128		A-Bba_J23100	

Supplementary Figures:

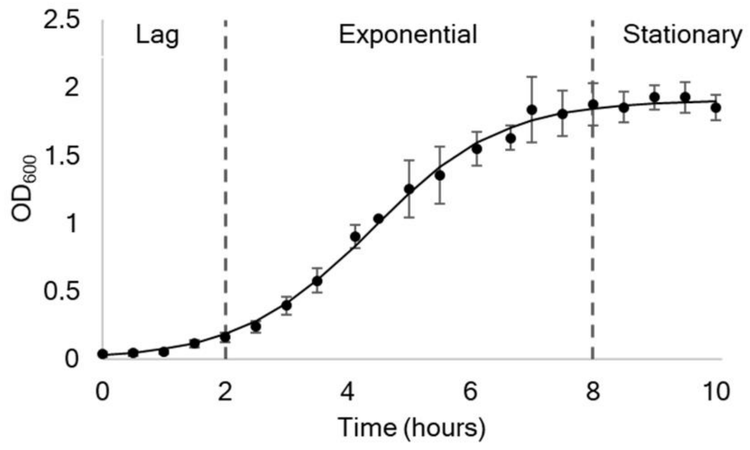

Figure S1. ***A. succinogenes* growth curve.** *A. succinogenes* growth curve with logistic model fit indicating the three growth phases; lag, exponential, and stationary. Error bars represent one standard deviation.

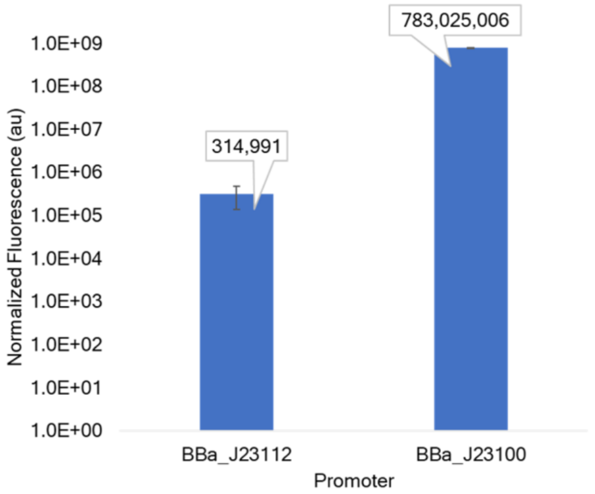

Figure S2. Expression in *E. coli* strain DH10B. GFPuv expression in *E. coli* strain DH10B with low and high expressing Anderson promoters normalized by absorbance and wild type (Materials and Methods). Error bars represent one standard deviation.

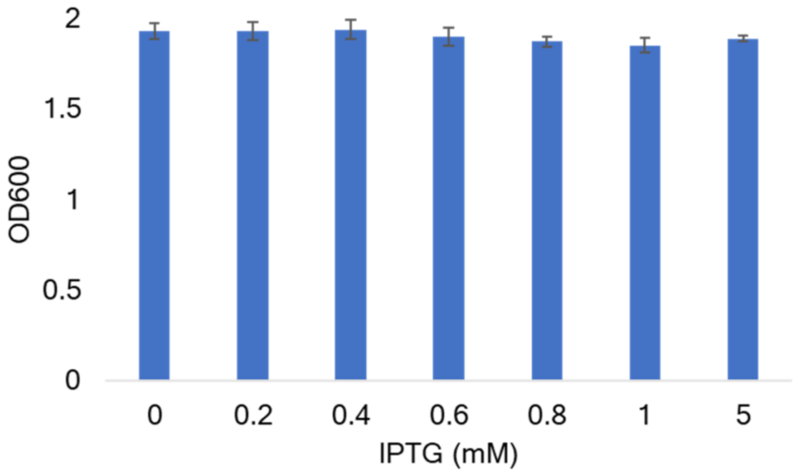

Figure S3. Induced A. succinogenes growth. OD_{600} of wild type *A. succinogenes* with varying concentrations of IPTG. Error bars represent one standard deviation.

Supplementary References:

1. Kim P, Laivenieks M, McKinlay J, Vieille C, Gregory Zeikus J. Construction of a shuttle vector for the overexpression of recombinant proteins in Actinobacillus succinogenes. Plasmid. 2004;51(2).
2. Kovach ME, Elzer PH, Steven Hill D, Robertson GT, Farris MA, Roop RM, et al. Four new derivatives of the broad-host-range cloning vector pBBR1MCS, carrying different antibiotic-resistance cassettes. Gene. 1995;166(1):175-6.
3. Lee TS, Krupa RA, Zhang F, Hajimorad M, Holtz WJ, Prasad N, et al. BglBrick vectors and datasheets: A synthetic biology platform for gene expression. Journal of Biological Engineering. 2011;5(1):12.
4. Immethun CM, Ng KM, DeLorenzo DM, Waldron-Feinstein B, Lee YC, Moon TS. Oxygen-responsive genetic circuits constructed in *Synechocystis* sp. PCC 6803. Biotechnology and Bioengineering. 2016;113(2).
